# Mapping Pharmacologically Evoked Neurovascular Activation and Its Suppression in a Rat Model of Tremor Using Functional Ultrasound: A Feasibility Study

**DOI:** 10.3390/s23156902

**Published:** 2023-08-03

**Authors:** Rohit Nayak, Jeyeon Lee, Setayesh Sotoudehnia, Su-Youne Chang, Mostafa Fatemi, Azra Alizad

**Affiliations:** 1 Department of Radiology, Mayo Clinic College of Medicine and Science, Rochester, MN 55905, USA; 2 Department of Neurologic Surgery, Mayo Clinic College of Medicine and Science, Rochester, MN 55905, USA; chang.suyoune@mayo.edu; 3 Department of Physiology and Biomedical Engineering, Mayo Clinic College of Medicine and Science, Rochester, MN 55905, USA; fatemi.mostafa@mayo.edu

**Keywords:** essential tremor, ultrasound, functional ultrasound, harmaline-induced tremor

## Abstract

Functional ultrasound (fUS), an emerging hemodynamic-based functional neuroimaging technique, is especially suited to probe brain activity and primarily used in animal models. Increasing use of pharmacological models for essential tremor extends new research to the utilization of fUS imaging in such models. Harmaline-induced tremor is an easily provoked model for the development of new therapies for essential tremor (ET). Furthermore, harmaline-induced tremor can be suppressed by the same classic medications used for essential tremor, which leads to the utilization of this model for preclinical testing. However, changes in local cerebral activities under the effect of tremorgenic doses of harmaline have not been completely investigated. In this study, we explored the feasibility of fUS imaging for visualization of cerebral activation and deactivation associated with harmaline-induced tremor and tremor-suppressing effects of propranolol. The spatial resolution of fUS using a high frame rate imaging enabled us to visualize time-locked and site-specific changes in cerebral blood flow associated with harmaline-evoked tremor. Intraperitoneal administration of harmaline generated significant neural activity changes in the primary motor cortex and ventrolateral thalamus (VL Thal) regions during tremor and then gradually returned to baseline level as tremor subsided with time. To the best of our knowledge, this is the first functional ultrasound study to show the neurovascular activation of harmaline-induced tremor and the therapeutic suppression in a rat model. Thus, fUS can be considered a noninvasive imaging method for studying neuronal activities involved in the ET model and its treatment.

## 1. Introduction

Essential tremor (ET) is one of the most common movement disorders and the most frequent pathologic tremors in adults, affecting 4–5% of the population over 65 years of age, with 60 million affected worldwide [1,2]. Currently, clinical diagnosis of ET has an estimated error margin of 37% of false positives [3]. Furthermore, treatment of ET focuses on pharmacological agents of various mechanisms, however, only ~50% of patients benefit from a particular agent [4]. To develop new medications, research on potential tremor-suppressing drugs in fully validated preclinical models of tremor is warranted [5]. Systemic administration of harmaline, a derivative of beta-carboline, is the most commonly used pharmacological approach to induce tremor in an animal model [5,6,7,8]. Furthermore, beta-blocker administration, commonly propranolol, which is the first-line pharmacological treatment of ET, is used as a suppressor of the harmaline-induced tremor in animal models [9,10]. Understanding the mechanisms of action of various tremor-suppressing agents is critical for future drug development. To investigate harmaline-induced brain activities, functional imaging studies play a crucial role. Neuroimaging methodologies could potentially improve the diagnostics by gaining insight into underlying brain pathology and could ultimately be used as a valid diagnostic tool [4].

Currently, the most frequently utilized imaging technique to study the pharmacological effects in an animal model is functional magnetic resonance imaging (fMRI), which shows regions of activity by using blood-oxygen-level-dependent (BOLD) signals [11]. However, one of the disadvantages of fMRI is its limited spatial resolution (~1–3 mm) [12]. Moreover, it is costly, noisy, and nonportable [13]. Breakthroughs in the field of high-frame rate imaging using plane wave ultrasound and advances in spatiotemporal clutter filtering techniques have dramatically improved the resolution of blood flow imaging [14]. Compared to conventional ultrasound, which can capture 50 frames per second, ultrafast ultrasound imaging can capture up to 20,000 frames per second [15,16]. Functional ultrasound imaging, a technique that has recently emerged, relies on ultrafast imaging scanners that provide high spatiotemporal resolution images of whole-brain cerebral blood volume (CBV) changes without using contrast agents [17]. Enhanced blood flow signal allows the detection of slow blood flow variations in small arterioles (down to 1 mm s^−1^) [17,18,19]. Monitoring the changes in the blood flow of these microvessels indirectly shows the neuronal activities proportional to the concentration of dynamic red blood cells in the regional volume [18]. Functional ultrasound with high spatial resolution and millisecond time can be an efficient alternative to BOLD fMRI for functional brain mapping. Thus, fUS may be a valuable imaging tool for understanding the function and connectivity of the brain in response to different types of stimuli in animal models of movement disorders [20]. Investigators have used fUS for mapping brain activities in various models [21,22,23,24,25], as well as in humans [26,27,28,29,30]. However, fUS has never been used for mapping the brain in the pharmacologically induced tremor model and its suppression.

In this study, we test the feasibility of using fUS imaging to characterize the brain tremor activity in a rodent tremor model induced by harmaline administration and its suppression by propranolol. To the best of our knowledge, this study is the first to use functional ultrasound for this purpose. Herein, we hypothesize that fUS imaging can enable visualization of brain activation and deactivation in response to pharmacologically induced tremor and its suppression in high spatiotemporal resolution. This can be useful in determining connectivity between various structures in different phases of tremor activity and suppression. Furthermore, the results obtained using functional ultrasound imaging will be compared with electrophysiology data. 

## 2. Materials and Methods

### 2.1. Animals

#### 2.1.1. Animal Handling

All experimental procedures in this study were conducted in accordance with the Guide for the Care and Use of Laboratory Animals in an AAALAC, International-accredited facility approved by the Mayo Clinic Institutional Animal Care and Use Committee. All authors complied with the ARRIVE guidelines. A total of 6 Adult Sprague Dawley rats (*n* = 6), all males weighing 250–400 g, were enrolled for this experiment. Animals were socially housed together. The cages were cleaned daily before feeding and sanitized weekly. The records of the cleaning schedule, temperature, and humidity were monitored daily. The animals had free access to food and water.

#### 2.1.2. Surgery and Tremor Induction

Rats were anesthetized by urethane (1.5 g/kg). The depth of anesthesia was checked by pinching the animal’s hind paw and a tail pinch. As soon as the animal was fully anesthetized, it was placed on a stereotaxic frame (David Kopf Instruments, Tujunga, CA, USA). Body temperature was maintained at 37.5° using a flat heating pad with a rectal thermal probe feedback system set. Before the craniotomy, the midline was marked on the skin to align the center of the transducer. Next, the craniotomy was performed to make a window 5 mm wide along the medial–lateral (ML) direction (Coordinates from +4 to −1 mm relative to bregma) and 12 mm long along the anterior–posterior (AP) direction (coordinates from +4 to −8 mm relative to bregma). Surgical procedures were achieved by leaving the underlying dura matter intact to prevent cerebral damage. For the simultaneous electrophysiological recording, two stainless-steel Teflon-coated microwires (0.013″ in diameter, A-M systems) were inserted in the primary motor cortex (M1; AP: 1.5 mm, ML: 1.9 mm, DV: 1 mm from the cortical surface) and the VL Thal (AP: −2.4 mm, ML: 1.9 mm, DV: 5.8 mm from cortical surface). A screw electrode for reference and ground was placed over the left visual cortex area. The connecting wires and electrodes were then fixed and insulated by the dental cement with two additional anchoring screws over the left hemisphere.

After the surgical procedure, a high-frequency ultrasound transducer was positioned over the craniotomy window using the stereotaxic frame along the sagittal plane at ML = 1.8. After stabilization (at least 30 min), the baseline recording of the fUS imaging session was started. After 30 min of baseline fUS imaging recording, 20 mg/kg harmaline hydrochloride (Sigma-Aldrich, St. Louis, MO, USA) was intraperitoneally (i.p.) administrated. The dose was dissolved in saline (1 mL) immediately before use. After an additional 20 min from the harmaline injection, propranolol (i.p. 20 mg/kg in 1 mL saline; Sigma-Aldrich) was injected into each rat. The propranolol solution was also freshly prepared before the experiment.

### 2.2. Data Acquisition

**Figure 1:**
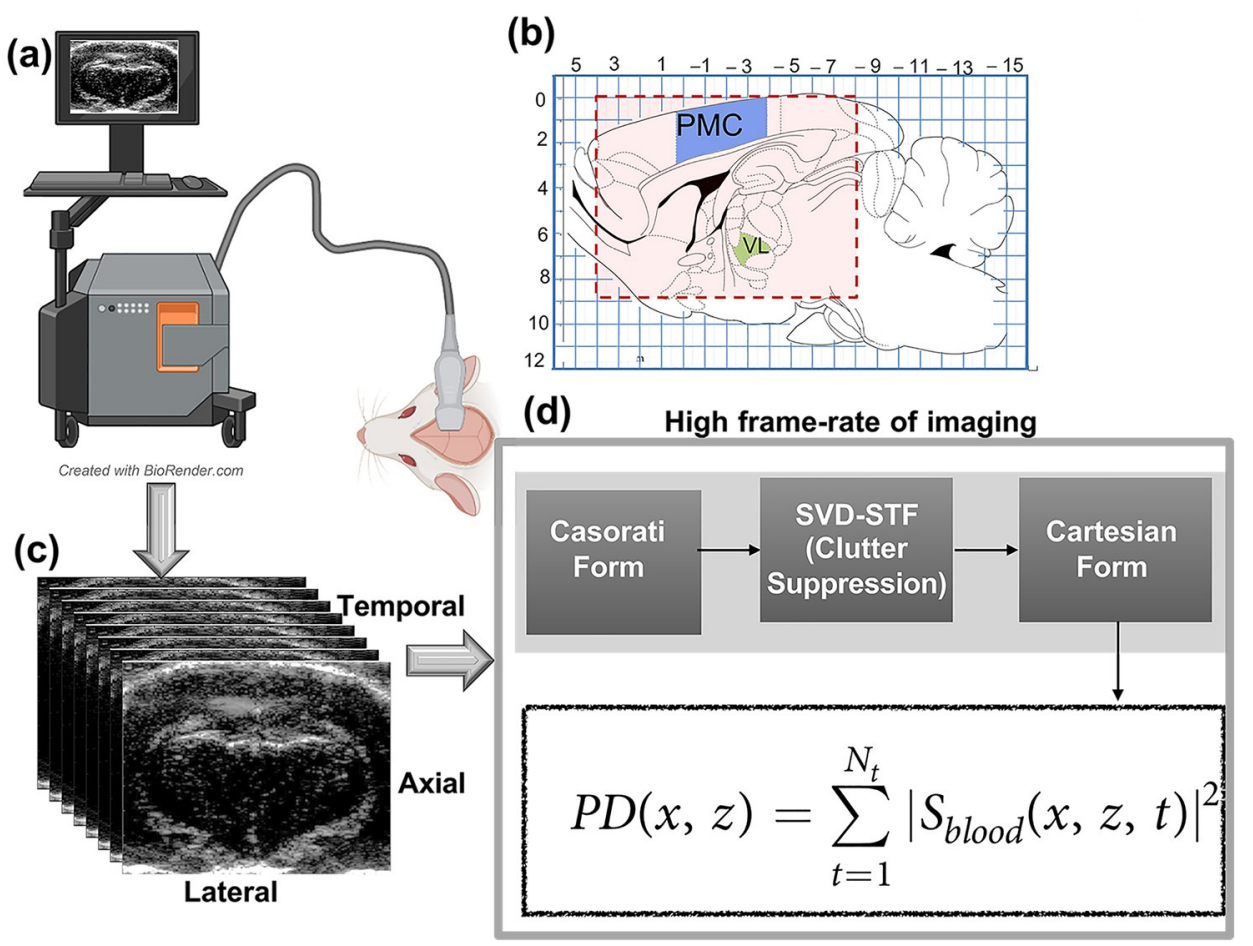
An illustrative depiction of the fUS experimental setup and imaging. (**a**) Ultrasound system and setup for the rat brain imaging. (**b**) A schematic of a sagittal cross-section of the rat brain corresponds to ML = 1.4 mm [1]. The regions of interest, PMC, and VL, are indicated in blue and green, respectively. The red ROI outlines the fUS imaging region considered in this study, (**c**) fUS image acquisition, and (**d**) steps of fUS image generation.

Ultrasound in-phase and quadrature (IQ) data were acquired using an ultrasound research platform, Verasonics Vantage 256 channel ultrasound machine (Verasonics, Kirkland, WA, USA). This research platform is equipped with a high-frequency 128-element linear array transducer L22-14 (Vermon, Tours, France). A thin layer of ultrasound gel was used to ensure acoustic coupling between the transducer and the rat brain. The ultrasound probe was placed directly over the cranial window, centered at the stereotaxic coordinate of ML: 1.4 mm. The position of the transducer was fixed for the entire study. For each microDoppler image, 400 IQ frames were acquired at a framerate of 1 KHz every 2.5 s at a transmit frequency of 16 MHz. The transmit sequence involved compounded plane-wave imaging at five insonification angles equally spaced between ±14°, with a pulse repetition frequency of 5 KHz. The received signal was beamformed using delay-and-sum. Figure 1a shows an illustration of the fUS imaging setup used for rat brain imaging. The rat brain schematic (Figure 1b) depicts the regions of interest and the overall imaging region. Figure 1c,d illustrates image and the steps for fUS image generation.

### 2.3. Functional Image Processing and Estimation of microDoppler Image

The acquired ultrasound Doppler ensemble was clutter-filtered using singular value decomposition to suppress the tissue clutter signal [14,31]. To separate tissue clutter from blood flow signal, a global singular order threshold was used. Each Doppler ensemble was reshaped into a 2D spatiotemporal Casorati matrix, and tissue clutter corresponding to the lower singular orders was rejected. Next, the tissue-filtered 2D Casorati matrix was reshaped into a 3D space–time Doppler ensemble. The clutter-filtered Doppler ensemble was coherently integrated to estimate the microDoppler image. The final microDoppler image was computed by coherently integrating the Doppler ensemble with time [32]. This process was repeated for all acquired Doppler ensembles. Furthermore, using denoising methods, the background noise bias in the microDoppler images was removed with respect to its clutter-filtered Doppler ensemble [33] to improve the visualization of the cerebral microvessels. The changes in cerebral blood volume (CBV) can be shown by functional activation maps (FAM) at any time instance, relative to the baseline. The FAM images were estimated by subtracting the time-averaged baseline signal from individual micro blood flow.

### 2.4. Electrophysiology Data Acquisition and Processing

Electrophysiological data were collected at 20 kHz sampling frequency using Intan amplifier (RHS 2000, Intan Technologies, Los Angeles, CA, USA). Furthermore, the data were down-sampled at 2 kHz and band-pass filtered from 0.1 to 500 Hz. A notch filter at 60 Hz with higher harmonics was applied to remove the potential line noise artifacts. The signals for three analytical time windows: T0 (20–30 min; before harmaline injection), T1 (35–45 min; immediately after harmaline injection), and T2 (60–70 min; immediately after propranolol injection) were selected. Using a multi-taper method (Chronux toolbox, version 2.12 v03** Latest release (25 October 2018)), the spectrograms of electrophysiology data were computed and normalized by an average baseline (0–20 min). All analyses were performed using functions in an EEG Lab (Delorme and Makeig, 2004), Chronux, and an in-house Matlab code based on previously published algorithms [34].

### 2.5. Statistical Tests

To assess the statistical significance of the difference in imaging response to HA-induced tremor and its suppression by Propranolol, the tremor activity was estimated in MC and VLT at specific time points. For instances of harmaline-induced tremor, functional activation at time points t = 30 min and t = 140 min, post-IP injections of the drug were considered. Furthermore, for harmaline- and propranolol-induced cases, time points of t = 30 min and t = 110 min were considered post-IP injection of Harmaline due to the delayed onset of the drug. Statistical significance was assessed based on the rank-sum test and computed in MATLAB, R2022a (The Mathworks Inc., Natick, MA, USA). for data points in the indicated ROIs corresponding to MC and VLT regions, at time points 30 and 120 min. A temporal averaging window of ±5 frames around the time points was considered to minimize the noise’s impact on the analysis.

## 3. Results

### 3.1. fUS Reveals Correlated Brain Activity Corresponding to Harmaline-Evoked Tremor

**Figure 2:**
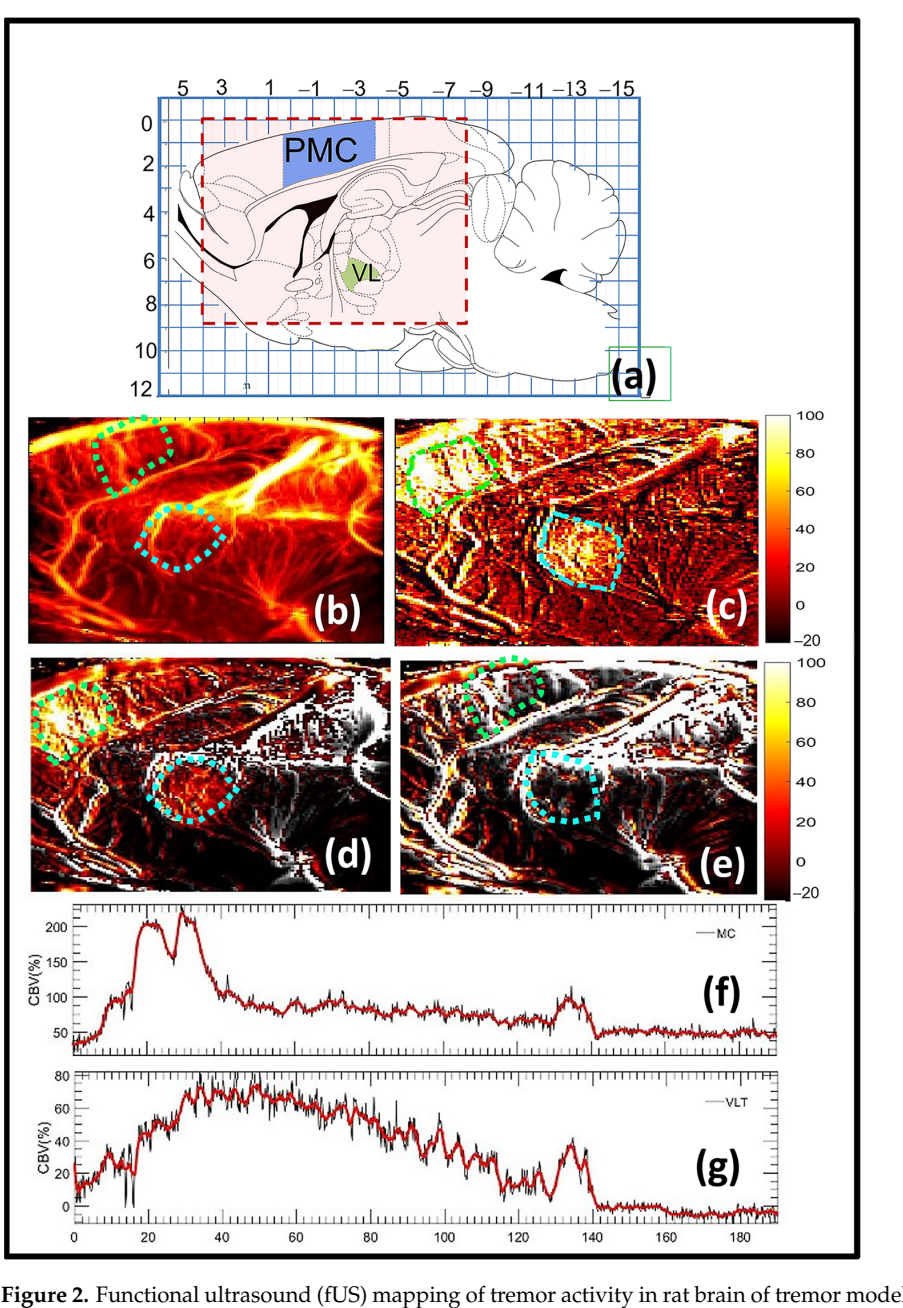
Functional ultrasound (fUS) mapping of tremor activity in rat brain of tremor model. (**a**) Rat brain atlas at the imaging cross-section of ML = 1.9 mm [35]. The motor cortex and the ventral lateral (VL) thalamus regions are identified in blue and green, respectively. (**b**) Rat brain microvasculature image. (**c**) fUS image 30 min post-IP injection of the drug, mapping the increased activation in the motor cortex and VL thalamus regions in light green and blue ROIs, respectively, (**d**) fUS signal overlaid on (**b**), depicting increased activation in the motor cortex and VL thalamus regions. The image corresponds to 30 min post-IP injection of the drug. (**e**) fUS signal overlaid on (**a**), depicting reduction in the motor cortex and VL thalamus activation after the drug effect subsides. This image corresponds to 180 min post-IP injection. (**f**) Mean of the fUS signal displayed in (**c**–**e**) in the motor cortex region as a function of time. The red plot indicates the mean of the fUS signals in the motor cortex area. The black plot displays a smoothed version of the red plot over a 1D kernel of 10 data samples. (**g**) Mean of the fUS signals displayed in (**c**,**d**) in the ventral lateral thalamus (VL) region as a function of time. The red plot indicates the mean of the fUS signals in the VLT area. The black plot displays a smoothed version of the red plot over a 1D kernel of 10 data samples.

The fUS imaging experiments were conducted on male adult, anesthetized rats with limited craniotomy. To understand how harmaline-related neuronal activity manifests through CBF activity in the rat brain, the contrast-free microvasculature images were acquired at 1000 Hz every 2.5 s. The fUS imaging constituted of 30 min of baseline recording followed by harmaline injection. Representative microDoppler images of rat brain across the sagittal cross-section (AP = 1.8), and the harmaline-induced CBF activity 30 to 140 min after the drug injection are displayed in Figure 2. The space and time-localized activation in the M1 and VL thalamus regions indicating harmaline-induced CBF alteration can be clearly observed in the functional ultrasound images (Figure 2c,d), which subsequently decays as the drug effect subsides with time (Figure 2e). Further, the CBF activity in the cortical region was limited to the primary and secondary motor cortex. The CBF increase in the thalamic region was specific to the VL thalamus. Further, the localized brain activity in the cortical and thalamic regions can be visualized in Figure 2d,e. The CBF increased over 160% upon harmaline injection, which gradually returned to baseline level as tremor activity subsided with time. This response was also corroborated through electrophysiology recording and behavioral study analysis. Line plots (Figure 2f,g) display the average change in blood flow volume (CBV) in the primary motor cortex and the VL thalamus, respectively, with respect to baseline, due to harmaline-induced tremor. The activation in the brain areas was observed to be highly localized to the motor cortex and VL thalamus areas, the former displaying the largest change in blow flow volume.

**Figure 3:**
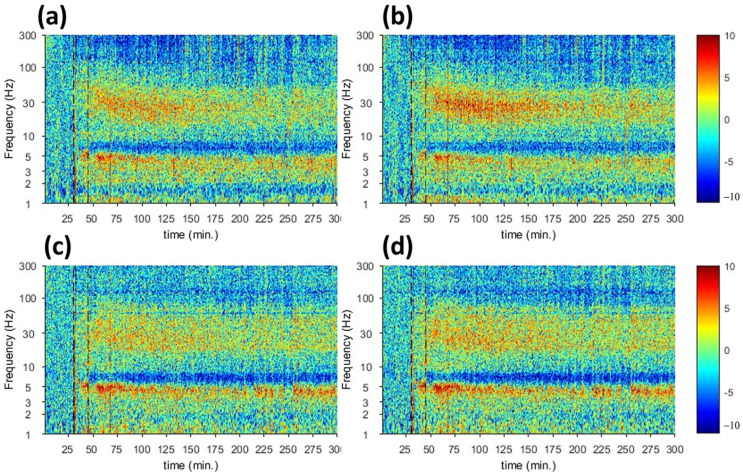
Spectral analysis of electrophysiology recording of tremor activity of rat brain in response to harmaline injection (The red dotted line shows the time of harmaline administration). (**a**) Motor cortex (M). (**b**) Ventral Thalamus (VlTh), (**a**,**c**) are left hemispheres and (**b**,**d**) are right hemispheres.

The spectral analysis of electrophysiology recording of tremor activity of the rat brain in response to harmaline injection is shown in Figure 3. The spectrogram of electrophysiology recording from M1 (Figure 3a,b) and VL thalamus (Figure 3c,d) in the left (Figure 3a,c) and right (Figure 3b,d) hemispheres. With respect to baseline reading, harmaline injection at t = 45 min displayed a significant increase in the spectrogram signal at approximately 5 Hz and 30 Hz in both cortical and thalamic regions, which gradually decreased with time.

### 3.2. Characterization of Putative Spatiotemporal Changes in Brain Activity Corresponding to Tremor Suppression Using Beta-Adrenergic Blocker

**Figure 4:**
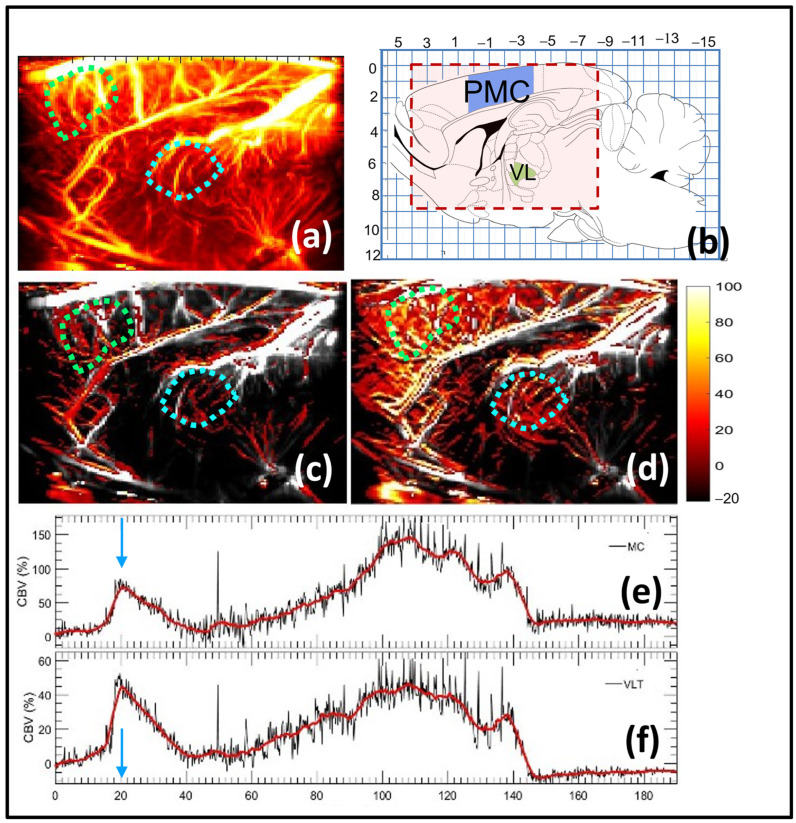
Functional ultrasound (fUS) mapping of tremor suppression in rat brain of tremor model. (**a**) Rat brain microvasculature image. (**b**) Rat brain atlas at the imaging cross-section of AP = 1.9 mm. The motor cortex and the ventral lateral (VL) thalamus regions are identified in blue and green, respectively [35]. (**c**) fUS signal overlaid on (**a**), depicting low activation in the MC and VLT regions, despite the injection of harmaline, due to propranolol effect. The image corresponds to 30 min post-IP injection of harmaline and 20 min after propranolol. (**d**) fUS signal overlaid on (**a**), depicting delay activation in the MCT and VLT regions. The image corresponds to 110 min post-IP injection of the drug. (**e**) Mean of the fUS signal displayed in (**c**,**d**) in the motor cortex region as a function of time. The red plot indicates the mean of the fUS signals in the motor cortex area. The black plot displays a smoothed version of the red plot, using a 1D kernel of 10 data samples. (**f**) Mean of the fUS signals displayed in (**c**,**d**) in the ventral lateral thalamus (VL) region as a function of time. The red plot indicates the mean of the fUS signals in the VLT area. The black plot displays a smoothed version of the red plot over a 1D kernel of 10 data samples. The motor cortex and the ventral lateral (VL) thalamus regions are identified in light green and blue ROIs, respectively.

We investigated the ability of fUS in demonstrating the effects of tremor suppressing drug (propranolol) on harmaline-induced CBF changes in an anesthetized adult male rat with limited craniotomy. The rats were administered with harmaline to modulate the tremor-related brain circuit, which was subsequently suppressed using propranolol, and injected 20 min later. The contrast-free microvasculature images were acquired in the sagittal plane at AP = 1.9 mm every 2.5 s (Figure 4a). The fUS imaging constituted of 30 min of baseline recording, followed by i.p. injection of harmaline (at 30 min) and propranolol for corresponding tremor suppression (at 45 min). The space and time localized activation in the M1 and VL thalamus regions can be clearly observed in the fUS imaging after the time point 15 min post-harmaline injection. However, a decrease in the harmaline-related activation was observed after propranolol injection, as observed at time point 45 min, which subsequently increased to demonstrate the effect of harmaline, i.e., time point 110 min. Figure 3b displays the rat brain atlas at the imaging cross-section of AP = 1.9 mm. Figure 4c displays the functional ultrasound image 30 min after harmaline injection (i.e., 10 min after propranolol injection), which displays a considerable decrease in the brain activity, followed by a gradual increase as displayed in Figure 3d. The brain activity observed in Figure 4d was limited to primary and secondary motor cortex and VL thalamus. Further, time plots of brain activity in these localized regions displayed a delayed onset of tremor. Line plots (Figure 4e,f) summarize the effect of both harmaline (at 0 min) and propranolol (20 min) injections, displaying a relative increase in the average blood flow volume (CBV) in the primary motor cortex and the VL thalamus, followed by a decrease due to propranolol, which subsequently regained activity owing to the residual harmaline effect. The activation in the brain areas was observed to be highly localized to the motor cortex and VL thalamus areas, the former displaying the largest change in blow flow volume.

**Figure 5:**
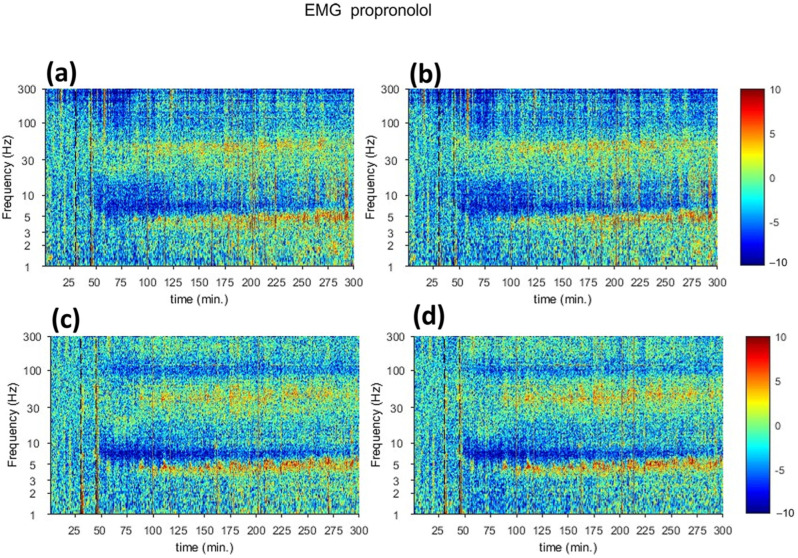
Spectral analysis of electrophysiology recording of rat brain in treatment response. (**a**) M1 (M), (**b**) VL Thal, (**a**,**c**) are left hemispheres and (**b**,**d**) are right hemispheres. The red dotted lines are the time points for harmaline and propranolol admiration, respectively.

This response was also corroborated through electrophysiology recording. Figure 5 displays the spectrogram of electrophysiology recording from the motor cortex (a,b) and VL thalamus (c,d) in the left (a,c) and right (b,d) hemispheres. In this experiment, suppression of harmaline-induced tremor is seen at t = 45 due to the injection of propranolol, and the gradual subsidization of anti-tremor effect of harmaline is noticed, causing an appearance of the STFT signals in both cortical and thalamic regions activities at t = 120 min.

Table 1 displays the comparison of harmaline-induced activities on MC and VLT of rat models of tremor for fUS and electrophysiology at two time points. Table 2 displays the comparison of the effect of propranolol in suppression of harmaline-induced activities on MC and VLT of rat models of tremor for fUS and electrophysiology at two time points.

### 3.3. Data Analysis

**Figure 6:**
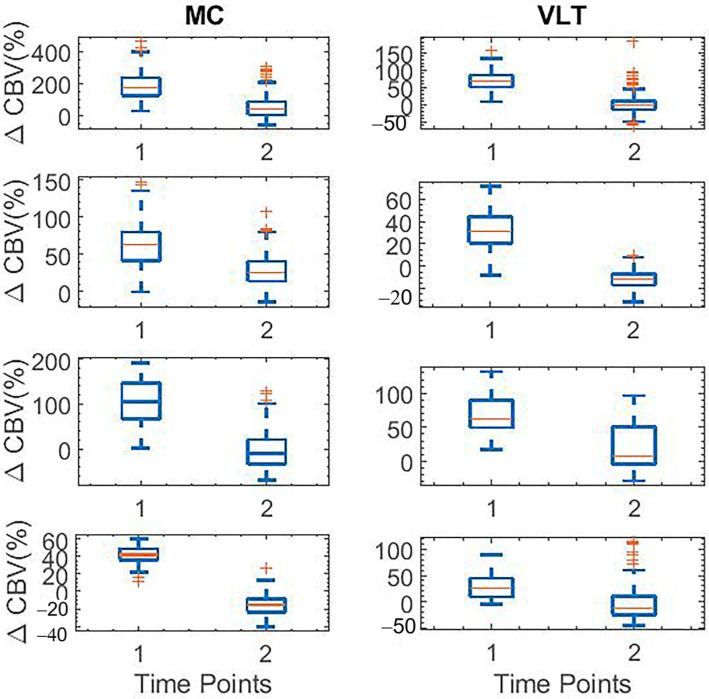
Boxplot data corresponding to the functional activation signal in MC (column 1) and VLT (column 2) regions in the rat brain at specific time points T1 and T2 post-IP injection of harmaline across four animals (rows 1–4). T1 = 30 min, T2 = 140 min. The percent change in CBV in MC and VLT was noted to be statistically significant (*p*-value < 0.01) across all animals. Each row in the box plot figure corresponds to an animal in the study. The red + symbols represent outliers.

**Figure 7:**
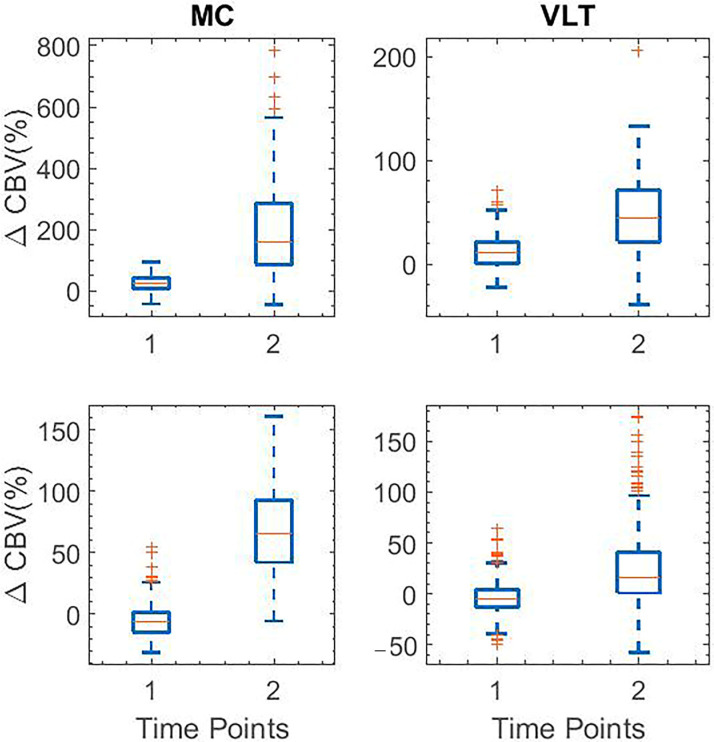
Boxplot data corresponding to the functional activation signal in MC (column 1) and VLT (column 2) regions in the rat brain at specific time points T1 and T2 post-IP injection of harmaline and propranolol across two animals (rows 1–2). T1 = 30 min, T2 = 110 min. The percent change in CBV in MC and VLT was noted to be statistically significant (*p*-value < 0.01) across both animals. Each row in the box plot figure corresponds to an animal in the study. The red + symbols represent outliers.

The box plot data in Figure 6 demonstrates the percent changes in CBV across all animals in response to harmaline, measured at the MC and VLT. Each row in the box plot figure corresponds to an animal in the study. Boxplots in column 1 correspond to the functional activation signal and in column 2 correspond to VLT regions in the rat brain at specific time points T1 = 30 min post-injection, and T2 = 120 min post-injection. Specifically, CBV was observed to be significantly (*p*-value < 0.01) higher at T1 = 30 min post-harmaline administration, which gradually subsided by T2 = 120 min. Boxplot data shown in Figure 7 corresponds to the functional activation signal in MC (column 1) and VLT (column 2) regions in the rat brain at specific time points T1 and T2 post-IP injection of harmaline and propranolol across two animals (rows 1–2). T1 = 30 min, T2 = 120 min. The percent change in CBV in MC and VLT was noted to be statistically significant (*p*-value < 0.01) across both animals. Each row in the box plot figure corresponds to an animal in the study.

## 4. Discussion

The current study demonstrates the feasibility of fUS imaging in characterizing cerebral hemodynamics in response to harmaline-induced tremor activities in rat model of tremor and its suppression after propranolol injection, which has an inhibitory effect on tremor. The spatial resolution of fUS using a high frame rate imaging enabled us to visualize time-locked and site-specific changes in cerebral blood flow associated with harmaline-evoked brain activity. Intraperitoneal administration of harmaline generated a significant CBF increase in the M1 and VL thalamus regions. The activities gradually returned to baseline level as tremor activity subsided with time. This response was also confirmed through electrophysiology recording; however, the peak for spectrogram signal arrives later than fUS. The temporal variation of tremor activation after the harmaline injection in our electrophysiology experiment was also consistent with the electrophysiology findings shown in [7]. The harmaline effect part of the experiment exhibits the potential of fUS in revealing brain activities in harmaline-induced tremor.

The inhibitory effect of propranolol was observed as a decrease in the tremor-related activation in cortico-thalamic regions. Our finding in this part of the experiment is evident that fUS can be considered a noninvasive imaging method for studying the development of therapeutic agents in animal models of tremor. These observations were confirmed by electrophysiological studies, showing a significant increase in the STFT signal in both cortical and thalamic regions after the Harmaline administration but no changes after harmaline and propranolol. However, the inhibitory effect of propranolol appears later in spectrogram than in fUS. Our findings are also consistent with the observations of the electrophysiological study indicating thalamocortical network’s involvement in harmaline-induced tremor [7].

The high prevalence of ET and the limited success in pharmacological treatment warrants the development of new therapeutic agents to achieve a better patient outcome. However, unavailability of validated preclinical models for evaluating potential tremor-controlling medications hindered the development of new effective medications. Harmaline-induced tremor is a suitable model for the assessment of effective tremor suppressant medications [10]. Moreover, functional neuroimaging is increasingly becoming an important tool for mapping activation signal of tremorogenic agent and its pharmacological response.

The emergence of fMRI techniques for brain mapping, based on the principle of blood-oxygen-level-dependent (BOLD) contrast changes, plays a valuable role in understanding movement disorders [36]. Investigators have used fMRI alone [37] or combined with electromyography [38,39,40] to study possible cerebral activation patterns in patients with essential tremor. Using resting fMRI, Wang et al. observed abnormal brain activities in the cerebello-cortical regions in hand and head tremor patients but in the cerebellar area in patients without head tremor [11].

Preclinical studies using fMRI endorse harmaline as a possible model for continued analysis of the brain regions involved in ET and its treatment. Our findings in demonstrating the activation associated with harmaline induced in agreement with the bold activation shown in [41]. A study by Lee et al. demonstrates significant activation of the olivo-cerebello-thalamo-cortical network associated with harmaline-induced tremor in a large animal model (pig), implying this network is a potential therapeutic target [41].

Low spatial resolution and limited availability of fMRI in animal facilities are the drawbacks of its use in preclinical studies. A recent breakthrough in ultrafast ultrasound technology has enabled substantial improvement in the sensitivity of slow and micro blood flow imaging resulting in the development of fUS to characterize changes in CBV in high spatial and temporal resolution [20]. Moreover, in fUS, the estimation of site-specific neuronal activity based on the assessment of CBV is potentially more sensitive than the BOLD signal of fMRI [42,43,44]. While the BOLD signal changes are related to the changes in CBV and O_2_ that can be affected by respiration rates, the fUS measurements are less impacted by the concentration of blood CO_2_ and O_2_, resulting in a more sensitive estimation of neuronal activities [44]. Therefore, employing the concepts of ultrasound and Doppler imaging, fUS can directly measure CBV changes in high sensitivity and spatial and temporal resolution.

Our study has some limitations. First, the sample size was small; future studies with larger numbers of rodents are warranted. Secondly, our experiments are conducted on anesthetized rats, and it is known that anesthesia may reduce cerebral activation due to its effect on neurological metabolism [45]. The future direction could advance the study of behaving animals, which may show a relative increase in overall cerebral response over anesthetized animals, as well as a better understanding of cerebral activation due to action tremors [46,47,48]. It should be noted that there is a potential for data degradation due to the motion in behaving animals, thus affecting the visualization of microvessels [49]. In future studies, one may employ motion correction algorithms to correct the degradations [32]. Thirdly, our fUS imaging was two-dimensional, as previously demonstrated [50]. FUS technology can be extended to three-dimensional (3D) imaging using matrix arrays connected to ultrasound machines with 1024 input-output channels. In future plans, one could advance fUS imaging to 3D fUS imaging technology for imaging cerebral response to pharmacologically induced tremor and its suppression in awake and behaving animals [26,51,52].

To the best of our knowledge, the current study is the first functional ultrasound study to show the neurovascular activation of harmaline-induced tremor and its pharmacological suppression in a rat model. Thus, fUS can be considered an imaging method for studying neuronal activities involved in ET models and its treatment. Further future investigations could include comprehensive studies using 3D fUS on larger numbers of behaving animals.

## Figures and Tables

**Table 1:** Harmaline-induced tremor in rat model.

	Moto Cortex	Ventrolateral Thalamus
fUS	Harmaline-induced activities start at t = 30 and decrease at t = 140	Harmaline-induced activities start at t = 30 and decrease at t = 140
Electrophysiology	Increase in the spectrogram signal at t = 45 and decrease in t = 145	Increase in the spectrogram signal at t = 45 and decrease in t = 175

t: time point.

**Table 2:** Harmaline and propranolol effect in rat model.

	Moto Cortex	Ventrolateral Thalamus
fUS	Suppression of harmaline-induced activities start at t = 30 and increase again with peak at t = 110	Suppression of harmaline-induced activities start at t = 30 and increase again with peak at t = 110
Electrophysiology	Low spectrogram signal at t = 45 and increase signal in t = 120	Low spectrogram signal at t = 45 and increase signal in t = 120

t: time point.

## Data Availability

The data that support the findings of this study are available from the corresponding author upon reasonable request. The requested data may include figures that have associated raw data. The request can be sent to: Karen A. Hartman, MSN, CHRC|Administrator-Research Compliance|Integrity and Compliance Office|Assistant Professor of Health Care Administration, Mayo Clinic College of Medicine & Science|507-538-5238|Administrative Assistant: 507-266-6286|hartman.karen@mayo.edu Mayo Clinic|200 First Street SW|Rochester, MN 55905|mayoclinic.org.m. We do not have publicly available Accession codes, unique identifiers, or web links.
